# Improving care engagement for mobile people living with HIV in rural western Kenya

**DOI:** 10.1371/journal.pone.0288087

**Published:** 2023-11-22

**Authors:** James Ayieko, Edwin D. Charlebois, Irene Maeri, Lawrence Owino, Marguerite Thorp, Elizabeth A. Bukusi, Maya L. Petersen, Moses R. Kamya, Diane V. Havlir, Carol S. Camlin

**Affiliations:** 1 Center for Microbiology Research, Kenya Medical Research Institute, Nairobi, Kenya; 2 Department of Medicine, University of California San Francisco, San Francisco, California, United States of America; 3 Department of Medicine, University of California Los Angeles, Los Angeles, California, United States of America; 4 School of Public Health, University of California Berkeley, Berkeley, California, United States of America; 5 Infectious Diseases Research Collaboration, Kampala, Uganda; 6 Department of Obstetrics, Gynecology & Reproductive Sciences, University of California San Francisco, San Francisco, California, United States of America; South African Medical Research Council (SAMRC) / Stellenbosch University (SU), SOUTH AFRICA

## Abstract

**Background:**

Antiretroviral therapy (ART) assures major gains in health outcomes among people living with HIV, however, this benefit may not be realized by all due to care interruptions. Mobile populations comprise a subgroup that is likely to have sub-optimal care engagement, resulting in discontinuation of ART. We sought to evaluate the barriers to care engagement among highly mobile individuals living with HIV and explore options aimed at improving engagement in care for this group.

**Methods:**

Qualitative in-depth interviews were conducted in 2020 among a purposive sample of twelve persons living with HIV and eight health care providers in western Kenya, within a mixed methods study of mobility in communities participating in the SEARCH trial (NCT01864603). We explored the barriers to care engagement among mobile individuals living with HIV and explored different options aimed at enhancing care engagement. These included options such as a coded card containing treatment details, alternative drug packaging to conceal drug identity, longer refills to cover travel period, wrist bands with data storage capability to enable data transfer and “warm handoff” by providers to new clinics upon transfer. Data were inductively analyzed to understand the barriers and acceptability of potential interventions to address them.

**Results:**

Stigma and lack of disclosure, rigid work schedules, and unpredictability of travel were major barriers to care engagement for highly mobile individuals living with HIV. Additionally, lack of flexibility in clinic schedules and poor provider attitude were identified as health-system-associated barriers to care engagement. Options that enhance flexibility, convenience and access to care were viewed as the most effective means of addressing the barriers to care by both patients and providers. The most preferred option was a coded card with treatment details followed by alternative drug packaging to conceal drug identity due to stigma and longer refills to cover travel periods.

**Conclusion:**

Highly mobile individuals living with HIV desire responsive, flexible, convenient and patient-centered care delivery models to enhance care engagement. They embraced simple health delivery improvements such as coded cards, alternative drug packaging and longer refills to address challenges of mobility.

## Introduction

At the end of 2021, an estimated 38.4 million [33.9–43.8 million] individuals were living with HIV, with more than two-thirds (25.6 million) of these in the African region [1]. Major milestones and advancements have been achieved in treatment and to a large extent, control of the HIV pandemic through scale up of Antiretroviral Therapy (ART) and aggressive prevention interventions. However, more needs to be done within the context of the double HIV/COVID-19 epidemic which has caused significant disruption to HIV care services [2]. To realize the proposed 95-95-95 UNAIDS targets aimed at ending AIDS by 2030, aggressive, targeted and deliberate efforts must be made by focusing on specific groups that suffer challenges in HIV care engagement [1, 3]. One such group is highly mobile individuals who have been shown to be susceptible to treatment interruptions [4].

The current HIV epidemic control strategy hinges on maximizing testing coverage, ART initiation, and achieving and maintaining viral suppression [5]. High levels of engagement in care and adherence to ART at population level for those living with HIV holds the promise of controlling the HIV epidemic through a strategy known as “treatment as prevention” [6]. However, geographic mobility is recognized as a key driver of the HIV epidemic in Africa [7–9] and a threat to care engagement [10] at all steps of the care cascade. Mobility has potential to destabilize care engagement and treatment outcomes in a context of living with HIV where, currently, individuals require daily oral pills to achieve viral suppression and lead normal lives [11]. The prevalence of viral suppression among adults in Kenya is estimated at 71.6% and varies from 39.7%-84.0% across different counties [12]. Mobile individuals in sub-Saharan Africa have been shown to be 1.3 to 2.6 times less likely to achieve viral suppression than their non-mobile peers [13, 14]; in Kenya and Uganda, mobility is associated with lower rates of HIV testing [15] and ART adherence [16], which likely leads to similar impact on viral suppression as seen in other settings. Viral suppression is crucial in prevention of onward transmission of HIV to sexual partners as demonstrated by U = U (undetectable equals untransmissible), further emphasizing the need to remain adherent to treatment [13–15, 17–19]. The mechanisms by which mobility leads to treatment interruption are not fully characterized, but qualitative studies suggest that unexpected travel and inflexible health systems can lead to missed appointments, and mobile individuals face many subsequent barriers to returning to care [20–23]. Though it is difficult to quantify mobility across diverse settings where different metrics are appropriate, mobility is a common phenomenon throughout sub-Saharan Africa, and is likely to increase in the future as a result of climate change, economic inequality, and conflict [23–26].

Deliberate measures must therefore be instituted by the healthcare delivery system to ensure optimum health outcomes for highly mobile individuals living with HIV by enhancing uninterrupted care engagement in spite of geographic mobility. The measures must generally be convenient, safe, feasible, acceptable, and effective in maintaining continued engagement in care with favorable health outcomes for people living with HIV as well as their partners. Though mobility is clearly a threat to adherence and health outcomes [10, 22, 27–29], much remains unknown about the specific challenges faced by mobile PLHIV and how mobility affects their engagement in ART. Even less is known about optimal solutions to these challenges and which interventions are most acceptable to highly mobile clients.

Within a mixed methods study of mobility in communities participating in a large cluster randomized trial conducted in rural East Africa (SEARCH trial), we conducted qualitative research to gain a deeper understanding of the challenges to care engagement among mobile individuals living with HIV. In interviews with mobile individuals living with HIV and health care providers, we sought to further ascertain preferences and assess potential feasibility and acceptability of a set of options for enhancing engagement from both patients’ and providers’ perspectives.

## Methods

### Study context

This analysis was conducted within the Mobility in SEARCH study (R01MH104132) [27, 30], a five-year study of mobility, risks of HIV and STI acquisition, and HIV care cascade outcomes in a cohort of adults in 12 communities in Uganda and Kenya participating in the Sustainable East Africa Research in Community Health (SEARCH) HIV test and treat trial (NCT#01864603) [31]. The study’s aims were to measure the complex forms of mobility of individuals in eastern African communities and to estimate the impact of that mobility on HIV incidence and on HIV care cascade outcomes. A longitudinal qualitative study was included which engaged community members in annual discussions of cumulative findings and their interpretation, including in year three discussions about possible services or interventions that could help to address challenges to care engagement among mobile persons living with HIV. In year four, this culminated in a community participatory rank choice voting exercise in each community, in which a set of options for possible interventions were prioritized by community members. This qualitative study was then conducted in a small sample of people living with HIV (PLHIV) in the study area in Kenya, to validate survey findings regarding the main barriers to care engagement among PLHIV, and further inform the development of intervention options to improve care outcomes in mobile PLHIV in a setting of high mobility. The goal was to culminate in development of options for interventions that would improve care engagement among mobile individuals.

### Study design and sampling

A purposive sample of participants balanced for gender was selected from six Kenyan HIV-care intervention clinics in the SEARCH HIV test-and-treat trial. The participants included twelve adult (> = 18 years old), mobile (spent >1 night in the last one month outside the community) patients already engaged in care and on ART and eight health care providers currently offering HIV care and treatment services to people living with HIV. The health care providers were comprised of five clinical officers and three nurses. Six of the interviewed participants living with HIV were female and six were male. Four of the health providers were female and four were male. Data were collected from January through February 2020.

### Data collection and analysis

Participants were contacted prior to the interview and the location for the interview was chosen by participants to suit their desired privacy, comfort and convenience. Data were collected using in-depth semi-structured interview guides. Trained qualitative researchers administered the interviews in the participants’ preferred language (English, Luo or Swahili). All study participants offered written consent. A common interview guide was used among the patient cohort and a different one for the provider cohort. Probes were added to the semi-structured guides as appropriate during the session to suit the different roles of the participants and seek clarity for responses given. Interview guides explored short- and long-term mobility, reasons for mobility and the effect of mobility on HIV care engagement (both adherence to treatment appointments and medications). The guide further prompted for reactions to five innovative options aimed at improving care engagement for mobile populations, exploring participants’ perceptions of the strengths and weaknesses of each option, and thoughts about ways of improving them. The options were described in detail as outlined below before participants were asked focused questions about each option:

1. Coded cards: a card provided to patients with coded treatment details including current regimen and home facility contact information to enable providers in any clinics that a patient attends to continue appropriate care engagement.
2. Alternative drug packaging: in contrast to standard bottle packaging of medications, pills would be packaged in paper envelopes, plastic bags, or blister packs for convenience and to mitigate stigma associated with ART packaging especially during travel.
3. Longer duration drug refill: mobile patients would be offered long refills at their home clinic during clinic visit to cover the periods patients anticipate to be away. This requires evaluation of patients’ travel plans/intentions at every clinic visit by the provider.
4. Wrist bands: a flash disk attached to a wrist band with treatment data including full treatment history, to be presented by patient at any clinic on arrival, and able to be updated at each visit.
5. ‘Warm handoff’: When patients relocate, providers from their home treatment facility would contact providers at the receiving facility to review treatment details for continuity of care engagement.

Pre-labeled cards with a picture of each model were displayed to the participant and explained individually. Participants were then given time to “internalize,” think through and ask questions on each option before identifying their preference and stating reason(s) for their choice. After participant’s choice of their most preferred model, other cards were kept aside leaving the most preferred choice for discussion. The remaining cards were then re-displayed for the participants to choose and discuss their models of choice in order of preference from the remaining options. The options were ranked based on patient and provider preferences as elicited during the interviews. These rankings were assessed separately for patients and providers. Preference was based on convenience for patient, ease of implementation for provider and potential challenges for both patients and providers. Finally, the participants were asked not only about how each option might be improved, but also to recommend other options, besides the five proposed, that would enhance care engagement for mobile individuals living with HIV. The interviews took approximately one and a half hours.

Audio recordings of interviews were transcribed and translated to English for analysis, which was conducted using a thematic approach [32, 33]. *A priori*, or deductive codes, were developed based on theory-informed interview guides. Subsequently, inductive codes were developed through focused coding of the empirical data. The coding framework generated was iteratively refined during data collection. Transcripts were coded using Dedoose Version 9.0.46. Four coders reviewed the first two transcripts independently to establish concurrence in themes and subthemes emerging from data. Subsequent transcripts were coded by one coder with frequent review of the code by two other members of the research team. In cases where new themes were emergent, these were discussed by the study team and included as new codes.

### Ethical approval

Ethical approval to conduct the study was received from the University of California, San Francisco Committee on Human Research (12–09555), Makerere University School of Medicine Research and Ethics Committee (2013–002), Uganda National Institute of Science and Technology and the Scientific Ethical Review Unit of the Kenya Medical Research Institute (KEMRI/CMR/RCTP/SSC 2453). All participants involved provided written consent to participate in the study.

### Inclusivity in global research

Additional information regarding the ethical, cultural, and scientific considerations specific to inclusivity in global research is included in the S1 File.

## Results

### Overarching themes

The purpose, duration, and frequency of travel varied between participants, notably between men and women, and travel was often unpredictable and unplanned. Mobility created conflict with ART engagement due to a variety of factors, including some specific to individuals such as lack of predictability of travel, the nature of their work (especially related to rigid schedules and incompatibility of work demands with clinics schedules), and their fear of disclosure, as well as health-system factors common to all clients such as inflexible refill schedules and providers’ attitudes towards mobility. Several of the care engagement options presented were acceptable and desirable, though participants wanted each intervention designed around the realities of clients’ mobility, specifically for convenience, practicality and confidentiality.

### Drivers of short-term mobility for diverse purposes

Participants described travel for economic gains, education, and religious and social reasons such as leisure, attending funerals and weddings. The duration of travel varied from days to months. Mobility was often influenced by “push” factors (undesirable influences that motivate people to move from their areas of residence) whose timing may be unpredictable.

“*…*.*I can’t say (predict) that I will travel to Homabay on this particular days… no*! *it depends with the customers and how I sell omena (fish)*.*”(28-year-old Female*, *Fish Vendor)*

We further observed a gender dimension to mobility, with women disproportionately experiencing limited control over their mobility and more adverse effects. For example, between the two genders, marital conflicts were more likely to move women out of their marital homes, and money (and by extension, power) in relationships was often controlled by men, leaving women with less control over their mobility.

“*(I had a case of a woman who I gave drug refills for)…six months and this client could not go back because the husband chased her away*, *so she came here to look for something to do as they were sorting their issues*. *It was not easy for her to go back (to her home clinic) due to lack of transport and up here she was also struggling to make ends meet with her children*.*” (Nurse*, *Humanist Clinic)*

Women also reported having multiple sexual partners, sometimes in different locations. Some of the partners were viewed as a source of sustenance: they supported income and gave favors such as providing lodging that reduced the cost of travel, and by this even determined the woman’s movement patterns.

“*(In the past 3 months) I went to Kisumu twice to meet my partner*, *he came from Bondo and we met in Kisumu twice and spent in a lodging…*. *mostly wherever I normally go to buy my stock I don’t use my money for paying lodging*. *Like in Busia there is S who caters for my lodging expenses*. *He is also another partner*. *It is in Nairobi where I have challenges when I travel for business*. *I can’t afford lodging when my casual partner is not around so I travel overnight*, *buy stock during the day and travel overnight coming back*.*” (45-year-old Female*, *Beauty shop owner and vegetable vendor)*

### Challenges to care engagement posed by mobility

It was observed that frequent travel often resulted in poor outcomes and patients being lost to follow up as they dropped out of care. The likelihood of disengagement was affected by factors specific to the individual including unpredictable travel, the nature of mobile employment, and stigma, as well as health system factors experienced by all clients including inflexible clinic schedules and providers’ attitudes.

*a) Lack of predictability*. Unpredictable and unplanned travel presented a major challenge in adhering to treatment. Among most participants, there were reports of not being able to refill their medications due to the abrupt nature of travel and of not remembering to carry their medication at all. To cope with forgotten medications, some individuals share drugs with friends. This may have resulted in mixing medication regimens.

Clinics were often not informed of unplanned travel and providers were unable to help patients plan. The lack of predictability of travel presented a great challenge in offering quality care to people living with HIV.

“*Sometimes they just leave abruptly*, *nobody tells you……They may only come back*, *three or four months later and would want to start again*, *so for them you can’t follow up on their viral load*, *you can’t follow up their good treatment plan because they are on and off care*.*” (Nurse*, *Humanist Clinic)*

Several providers noted fishermen were rarely in control of their movements, relying instead on boat owners’ decisions regarding fishing locations, and were therefore unable to plan for medication refills.

*As a fisherman I move to several places like Sindo*, *Uyoma*, *Sakwa and others*. *I can stay there for about a month*. *[My travel] is guided by the difficulties around here especially when the catch is low*. *I don’t usually plan to travel in which specific times of the year*. *We only move based on the rumors of the fish availability in those other places… There are [also] very many unplanned travels like for funeral or just visiting a friend*. *(36-year-old Male*, *Fisherman*)

If participants did try to access care in a new location unexpectedly, they may be hampered by not knowing their ART regimens.

“*Sincerely I don’t know the names of all the regimens… septrine*, *lamivudine I don’t know what*. *If the card is lost and I need a refill at a facility other than Ogongo*, *then I am doomed*.*” (45-year-old Female*, *Beauty shop owner and vegetable vendor)*

*b) Nature of work*. Some situations specific to certain occupations such as staying in the lake for prolonged periods made it difficult to adhere to care, with some clients missing clinic appointments as well as medications if their drug supplies were not refilled. Employment conditions for those who have moved looking for employment opportunities made it difficult to engage in care, especially if employees did not have freedom to attend clinic on their allocated appointment dates.

“*I used to work for someone in Mariwa*, *he was a very difficult person and we could work for seven days in a week*. *I did not even find it easy to ask for permission to go to the hospital and that lead to dropping out of care for about two years*.*” (30-year-old Male*, *Truck driver)*

The nature of mobile employment also made it more difficult to cope with side effects. Fishing and preparation of fish before sale, two activities often conducted by mobile clients, were especially difficult when suffering drug side effects such as dizziness.

“*I normally take my drugs at night*, *but this particular one whenever I am going to the lake to look for fish*, *I have to take it in the morning*. *I normally deep-fry fish in the night and whenever I take this drug at night I feel dizzy hence it interferes with my work*.*”(40 year old Female*, *Fish vendor)*

*c) Stigma and lack of disclosure*. In some instances, the drugs were left behind deliberately or pills not taken due to fear of disclosure of status due to stigma.

“*He (the patient) wasn’t using the drugs*, *he could take medication (from clinic) leave it at home and then go to (boarding)school*, *because he could not disclose to the school nurse and also to the fellow students that he is taking medication*.” (Nurse, Humanist Clinic, LO)

“*Mobile patients really have challenges because they fear others noticing that they are on HIV medicines*. *Sometimes they have even carried the drugs (during travel) but fear what others are going to say when they discover that s/he is on ARVs*.*” (39-year-old Female*, *Businesswoman and community health volunteer)*

Within the fishing community, characterized by high mobility, the effects of stigma and lack of disclosure were exacerbated. Participants described a high rate of relationship dissolution and change of partners, meaning PLWHIV experienced the fear of disclosing to a new partner more often. Many participants noted that this compromised care engagement further.

“*[Non-disclosure between partners] is very common along the beaches because at the beaches people marry today and separate tomorrow… (Laughter)*. *A woman will live with this fisherman; when he leaves for another beach*, *a new one replaces him*, *and probably they are both on ARVs but they can’t disclose this to one another because if she discloses*, *she will not get another temporary husband to meet her needs*. *Same to the fisherman*. *Those are cases that are very common at the beaches*.*” (39-year-old Female*, *Businesswoman and Community health volunteer)*

*d) Inflexible appointment schedules*. It emerged that failures or inefficiencies within the health care delivery system were a barrier to care engagement for mobile populations. Some of the issues described may have affected all clients, but their effect was magnified for highly mobile clients. Rigid clinic schedules were particularly not accommodative to individuals with travel plans, and providers reported that many clients missed appointments due to travel. Several participants described conflict that would ensue if they missed appointments: for example, if they arrived at a facility in the afternoon (often after a long journey to reach the facility), they would be turned away and told to return the next day, or if they missed an appointment entirely, they would be forced to attend “adherence classes,” even if the cause of the missed appointment was out of their control.

“*I dropped out of care for some time which even created a rift between me and the providers when I finally showed up to continue care again*.*” (30-year-old Male*, *Truck driver)*

“Sometime back there was a hitch when the date and the day indicated on my card clashed. I reported on the very date indicated and they claimed that I ought to have reported three days earlier. They argued that I was already a defaulter and had to be taken to defaulter class and I bitterly argued with them about it.” (46-year-old Male, Fisherman)

*e) Provider attitudes*. Mobile clients were especially vulnerable to poor relationships with providers. In addition to conflict over missed appointments, lack of confidentiality was a pronounced concern for clients who were new to their community and felt their social position was particularly precarious; unwanted disclosure may have had larger consequences for them. Fear of disclosure led to a lack of trust in the healthcare system, magnifying a perception that the health system was not supportive nor sensitive to the patients they served.

“*That is why some people drop (out of) care*. *S/he can say that providers are publicly announcing their HIV status to other people*. *Somebody will say ‘I will not go to that facility where my HIV status had already been announced*.*’ So they intentionally default… ‘these providers publicly announce people’s status to other facilities’*.*… People fear being exposed*.*” (39-year-old Female*, *Businesswoman and Community Health Volunteer)*

### Preferences for mobile care engagement options

Participants were presented with five innovations designed to improve ART adherence for highly mobile people. Of the options discussed with participants, the coded card was most preferred followed by the longer refills and alternative drug packaging. Participants also shared their recommendations to improve the options presented (Table 1).

**Table 1:** Care engagement options; patients’ and providers’ perspectives on pros and cons of each option and recommendations for improvement.

Care Engagement Option (Most-Preferred to Least Preferred)	Pros	Cons	Recommendations
**1. Coded Cards**			
Patients	• Convenient	• Risk of inadvertent disclosure of HIV status • Risk of loss is high • May be damaged by water • One may forget to carry card	Should be: • Water resistant • Small in size • Include name • Different colors
Providers	• Easy to implement • Offer correct regimen for refill • Patient can access care anywhere	• Risk of inadvertent disclosure of HIV status • Risk of loss is high • May lack detail required for management (e.g. viral load)	• Different colors • Add treatment details such as regimen, date of diagnosis, viral load
**2. Alternative Packaging**			
Patients	• Curtails noise made by bottles that discloses HIV status • Can carry smaller amounts of drugs to cover shorter periods	• Loss of drug potency • Water damaging drugs	• Should have water resistant packaging
Providers	• Helps address stigma associated with bottles • Boost patient esteem and confidence	• Loss of drug potency	• Add desiccant to alternative packaging
**3. Longer refills**			
Patients	• Minimizes visits to clinic	• Loss of drugs may be difficult to explain to provider	• Should not be too long because clinic visits serve other functions (e.g. social support)
**Providers**	• Reduces clinic visits • Reduces work load • Decongests clinics	• May not work well for unsuppressed patients • Stock outs by supply chain may hamper this option • Patients may forget clinic appointments • Poor drug storage may lead to potency loss	• Educate patients on drug handling and storage
**4. Wrist Bands**			
Patients	• Convenient • Ability to travel with all treatment details to enable continuity of care	• Risk of inadvertent disclosure of status • Risk of damage • Loss of wrist band • Loss of information • Others accessing patient information (lack of confidentiality) • Requires computer and power	• Different colors of wrist bands to avoid inadvertent disclosure
Providers	• Specific groups may have stronger preference (e.g. younger patient may find it stylish) • More convenient than paperwork • Patient information is likely to be complete	• Susceptible to damage by water • Risk of inadvertent disclosure of status • Expensive • Data can be altered • Requires computer and power	• Use different colors and designs • Encrypt data or use other security features to avoid data alteration • MoH to invest in infrastructure (e.g. power, computers)
**5. Warm Hand Off**			
Patients	• Detailed transfer of patient data for continuity in care	• Providers may reveal patient status to others	
Providers	• Can easily be standardized across clinics	• Additional workload • Time consuming, “hectic” • Depends on attitude of clinic and providers • Contacts for other facilities may not be available	• Standardize procedure of data sharing across sites

The card was considered convenient and private, without the risk of inadvertent disclosure (so long as the information was fully coded). Providers were eager to have access to information presented on the card but did have concerns that the card could fit all necessary details.

“*The good thing about it is that the patient will receive care at any point and because it is just a card it is easier to walk with*, *it can fit in a wallet or a lady’s handbag*. *It is coded*, *therefore it is only the provider who can understand what is in the card*. *In case any other person comes across it*, *s/he may not know what that card entails*.*” (Clinical Officer*, *Yokia Clinic)*

Alternative packaging was a popular option as well due to stigma associated with ART packaging, though nearly all participants expressed concern about the risk of damage to the medication.

“*I don’t like the noise that the bottle makes*. *I can also be very free with exposing my drugs (while using the envelope [alternative packaging]) because nobody will judge me about my HIV status*. *They will not know the kinds of drugs I am taking unlike the ARVs bottle*. *Even if I put the envelope on the table*, *nobody will know what drugs they are*.*” (51-year-old Female*, *Fish vendor)*

“*Alternative package can be very good*, *there was a time I went to Oresi and there was a patient who refused to take the drugs saying that she can’t carry the drugs packaged in a bottle because the bottle will make noise in her bag while in a matatu*. *Another patient said that she even fears using a motorbike when carrying the drugs because motorbike is worse with the noise from the bottle… ‘tuku tuku tuku tuku’…*. *(Laughter)*.*”* (*28-year-old Female*, *Clothes vendor)*.

“*…*. *drugs are not supposed to be removed from the bottle because of drug potency*. *That there is something put inside the bottle to preserve them*. *So when it is removed and put in an envelope or another package then it loses its potency*.*” (40-year-old Female*, *Fruit vendor)*

Both patients and participants saw the benefit of longer refills. Most providers associated longer refills with stable, virally suppressed clients, and were therefore unsure if they would be successful for highly mobile clients, but a few identified that longer refills may in fact make achieving viral suppression easier for mobile clients.

“*Most of them love longer refills but we only do them for specific group of patients like the ones that are suppressed*. *The virally suppressed are the only ones who get the chance to have drugs for six or three months*. *They appreciate that and the suppression continues*. *If we introduce that to the non-suppressed*, *I think we will mess things up*. *I think we just continue with the suppressed*.*” (Clinical Officer*, *Nyamrisra Clinic)*

Patient and provider preferences were largely similar. However, providers appeared to prefer clinic-based options such as longer drug refills as opposed to communicating with other providers. Both patients and providers expressed concerns about provider attitudes hindering options such as warm hand offs and viewed it as time-consuming.

“*Actually longer refills have minimal work load…laughter… because if I give you three months the next time I don’t use your file and that is faster for me*.*” (Nurse*, *Humanist Clinic)*

“*… the attitude of the providers might not make this option [warm handoff] work very well*. *In our facility we have a very bad provider*, *just in case she is the one who receives this call or the reverse of this*, *she is the one who is giving my information to the next provider … ‘the patient is M*, *she has been a bother to us*, *I don’t even know whether you will manage her*.*’ That alone will dampen the spirit of the other provider and I may not be received well in the other facility*.” (*45-year-old Female*, *Beauty shop owner and vegetable vendor*)

“*I might be willing to help but what of the other clinician the other side*, *you can’t be sure of the possible response that you are going to receive*. *It’s just pegged on hopes that you are likely to get a positive response and you are able to facilitate (transfer of patient details)*.*” (Nurse*, *Humanist clinic)*

On other options to enhance care engagement among mobile individuals living with HIV beside those presented, some participants suggested the option of long-acting injectable drugs as a preferred choice to care engagement.

“*I just want to ask if there is a possibility of availing injectable ARVs in future……I sometimes heard that injectable ARVs are supposed to be introduced*. *Is it still a valid thing*?*” (32-year-old Male*, *Miner)*

Providers went further to suggest a mobile clinic specifically to attend to mobile individuals living with HIV as well as unique identifiers for patients to enable seamless access to treatment at any clinic.

“*Maybe mobile clinics but that would require a lot of resources and monitoring patients’ viral load might become problematic*. *Probably… providing unique identifiers*… *So if a patient comes with that unique number to your facility then you obviously know (where) the patient is coming from and contacting particular facilities in that sub-county becomes easier in cases of follow ups to patients who don’t have contacts of primary clinics*. *Or*, *provide a unique number for mobile patients that providers are aware of and once they come to the facility they can easily be identified as patients on transit*.*” (Clinical Officer*, *Yokia Clinic)*

In summary, patients and providers are open to care engagement options that may address the conflict between mobility and ART, with a preference for coded cards, longer refills and alternative drug packaging. However, for each of the options, they described the realities of mobility–such as exposure to the elements, fear of poor provider treatment, and fear of disclosure–that should shape any subsequent intervention.

## Discussion

This study conducted among mobile individuals in rural western Kenya revealed that mobility remains a challenge to HIV care engagement. Mobility created conflict with ART engagement due to a variety of factors including lack of predictability of travel, the nature of work and its incompatibility with clinic schedules, and fears of disclosure, as well as health-system factors common to all clients such as inflexible refill schedules and providers’ attitudes towards mobility. Individual, socio-cultural and health system factors serve to complicate HIV care engagement in the context of mobility that is largely unplanned and unpredictable. Clinic schedules, provider attitudes, stigma and the inherent pressures of unpredictable travel all contributed to tension between their travel and ART adherence, confirming our earlier survey findings on barriers to engagement among PLHIV [34] while offering deeper insights on the mechanisms of the barriers.

We observed that in our study settings, travel was rarely undertaken purely for leisure: mobility is a livelihood strategy, and whether traveling for fishing, trading, caregiving, or to maintain family ties, mobility provided income and ensured social support for participants living in economically precarious situations [22]. A recent study on HIV care retention conducted in South Africa revealed mobility as the leading cause of care interruption for people living with HIV [4]. Unique patient-level as well as health system-level aspects make care engagement difficult for mobile individuals living with HIV. Our results on the barriers to care engagement are comparable with those of previous studies [28, 29], which found HIV-related stigma at travel destination, fear of disclosure and health system inflexibility with regard to dispensing medication are major impediments to care engagement by mobile PLHIV. To enable PLHIV to live full lives, the health system must adjust to accommodate mobility, rather than appear to discourage it or force clients to choose between ART and their livelihoods or social connections. By triangulating information from both patients and healthcare providers, this study revealed that flexibility, patient-friendly attitudes among health workers, and a client-centered health delivery system, shown to be highly effective in achieving patient satisfaction in other health care settings [35, 36], would similarly yield better outcomes among mobile PLHIV.

Our analysis revealed a gender dimension to mobility and, by extension, challenges in HIV care engagement. Men and women experienced different types of mobility, but both often had limited agency to control their movements; fishermen were beholden to boat owners, for example, while women’s movements depended in part on their partners who funded their travel. The fishing industry along the shores of Lake Victoria is centered on the Nile Perch and the Silver Cyprinid locally referred to as *omena*. These are migratory, and fishermen, for the requirement of maintaining their family livelihoods, follow the fish and move to trade along the beaches around areas with highest catch. Fish vendors at the beaches, mostly women, may also move to areas with highest catch for trade. This gendered dimension to mobility observed in our study aligns with findings from prior work by Camlin et al. [30, 37]. Women’s care engagement was more likely to be disrupted by relationship dissolution which forced them to move from their marital homes, a common challenge in patrilineal marriage systems [37] where the power-dynamics are more in favor of the man. The home is viewed as “belonging” to the man and so during marital conflict, the woman is forced to leave. This results in greater disruption in livelihood as well as health seeking engagements like HIV care among women than men in such instances. While interventions for highly mobile men and women might differ, we did not identify obviously different themes in their preferences for the care engagement options presented. Previous research among mobile populations has suggested that that understanding a combination of factors, such as seasonality, duration, patterns of mobility and characteristics of the mobile individuals may be more useful in identifying and targeting interventions to improve patient outcomes, rather than focusing on a singular characteristic [30].

Our study showed clear preferences for specific options among patients and providers. These largely appeared similar among both patients and providers, though for different reasons. While patients placed a high premium on confidentiality and convenience, providers appeared to prefer options that would reduce their workload demands while ensuring continuity of quality care for the patients. No single option was viewed as addressing the full range of challenges experienced by mobile persons. We observed much agreement in pros and cons between patients and providers in their assessment of the options provided with helpful recommendations on improvement of the proposed options being made by both groups.

A multi-pronged approach may be needed to address mobility-related barriers to care, with different stakeholders (policy makers, providers and patients) having flexibility to make adjustments to service provision and center service delivery on the patient. Health care systems must be deliberate in retention of “challenging” groups because care disengagement has been shown to occur over time, following a chain of events that can culminate in disengagement from care [20]. Care disengagement often results from the accumulation of multiple barriers, including mobility, but a determinative event may be as simple as a negative experience with a provider. There may be value in having providers go through training on improving treatment care for mobile patient populations as opposed to “forcing” mobile patients to attend adherence classes whenever they miss a clinic visit due to mobility. Several care engagement options were viable and acceptable, and patients may need multiple options to enhance engagement. Longer refills, or “multi-month dispensing,” has been highly effective in other contexts in reducing the burden of ART clinic visits for stable clients [38–40]; though it is usually restricted to clients at low risk of treatment interruption, mobile clients may indeed benefit from this care model. There is less data available regarding the other interventions proposed in our study, including coded cards, wrist bands, alternative packaging, and warm handoffs for mobile clients. Choice has been shown in previous studies to enhance outcomes in other areas such as HIV testing [41]. A streamlined, patient-centered approach has also been shown to improve patient outcomes across multiple points along the care cascade [42, 43]. These considerations need to be factored in engagement of highly mobile individuals living with HIV. Providers and patients want new innovations to be designed around the reality of mobility. Given the varied types of mobility observed in our study, it may be that different clients choose different interventions to meet their needs and switch their choice of options from time to time as defined by their circumstances.

Our study was not without limitations. The study may have been limited by the relative success of its patient participants; the participants included in the study were all currently engaged in care and clinically stable, with some reporting never having experienced treatment interruption. Those who are disengaged from care may face unique barriers and have preferences different than those included here. Subsequent studies should deliberately include mobile clients who have dropped out of care to best identify care engagement options that may have supported them more effectively. Secondly, this study does not provide data on the extent and different types of mobility in the overall patient population and therefore is unable to give specific insights on the different categories of mobility. Finally, there is a trade-off between open-ended questions about what kinds of interventions would help clients most versus close-ended questions presenting specific options for care engagement; by presenting specific options, this study may have limited participants from identifying and sharing other innovation options to improve care engagement among mobile individuals. On the other hand, the study was strengthened by its inclusion of both patients and providers. These two perspectives presented us with an opportunity to triangulate our findings. The diverse perspectives complemented and confirmed each other’s viewpoints while adding nuance to our understanding of mobility and its relationship to care engagement.

## Conclusion

The health care system needs to be sensitive and responsive to the needs of highly mobile individuals living with HIV. A supportive policy framework that addresses the barriers to care engagement will make a great impact in enhancing care engagement among this group. Mobile individuals embraced simple health delivery improvements such as coded cards, alternative drug packaging and longer refills to address challenges of mobility. These options were viewed as feasible and generally acceptable among both patients and healthcare providers. Further studies are required to scale up these proposed care engagement options and evaluate patient uptake, utilization and retention with resultant viral suppression outcomes.

## Supporting information

S1 File Inclusivity in global research. Details on conduct of the research.
(DOCX)
