# Supplementary material for: Improving care engagement for mobile people living with HIV in rural western Kenya
Source: PLoS One. 2023 Nov 22;18(11):e0288087. doi: 10.1371/journal.pone.0288087 (PMC10664942; doi:10.1371/journal.pone.0288087)
Supplement: S1 File — Details on conduct of the research. (DOCX) [file pone.0288087.s001.docx]

S1 Checklist: Inclusivity in global research

PLOS’ policy on inclusivity in global research aims to improve transparency in the reporting of research performed outside of researchers’ own country or community and ensures that PLOS publications reporting global research adhere to high standards for research ethics and authorship.

**Ethical considerations, permits and authorship**

*This section is applicable to all research types.*

Provide details as to who granted permissions and/or consent for the study to take place in the Methods section of your manuscript. This should include the names of **all** ethics boards, governmental organizations, community leaders or other bodies that provided approval for the study. If individuals provided approval refer to these people by their role or title but do not list their name(s).

Reported on page number: 9

If there were any deviations from the study protocol after approval was obtained please provide details of these changes in the Methods section of your manuscript.
Did this study involve local collaborators that are residents of the country where the research was conducted or members of the community studied? If you do not have any authors from said communities, please provide an explanation for this below.

Reported on page number: 9

Study involved local collaborators. I(JA), the lead author is a Research Scientist at the local Kenya Medical Research Institute and a resident of Kenya where the study was conducted.Other local collaborators from Kenya are IM,LO,EAB.

Everyone listed as an author should meet PLOS’ criteria for authorship and all individuals who meet these criteria should be included in the author byline, rather than the acknowledgements. Authorship criteria is based on the International Committee of Medical Journal Editors (ICMJE) Uniform Requirements for Manuscripts Submitted to Biomedical Journals - for further information please see here: <https://journals.plos.org/plosone/s/authorship>.

**Human subjects research (e.g. health research, medical research, cross-cultural psychology)**

Did you obtain written informed consent from a representative of the local community or region before the research took place? How did you establish who speaks for the community? Details of written informed consent obtained from study participants should be reported separately in the Methods section of your manuscript.

Our community entry comprised holding meetings with the leadership of the community, the administration at the Subcounty and the village level. We constituted a community advisory group that gives feedback and helps define the conduct of the study in the areas where we coduct the study. For the clinic, we recived approval from the clinic-in-charges to involve providers in the study.All participants were signed a written informed consent before participating in the study.

How did members of the local community provide input on the aims of the research investigation, its methodology, and its anticipated outcome(s)?

After approval of the study by the local IRB, we constituted a community advisory group comprised of members of the community that evaluates what the study aims to do. This groups helps in defining the conduct of the study by providing feedback and suggestions on how to conduct different aspects of the study.

When engaging with the local community, how did you ensure that the informed consent documents and other materials could be understood by local stakeholders?

We had the informed consent in three languages; Luo(local language), Swahili(national language) and English. Participants were allowed to select the language they are most comfortable with to ensure understanding before consenting to participate in the study.

Will the findings of the research be made available in an understandable format to stakeholders in the community where the study was conducted (e.g. via a presentation, summary report, copies of publications, etc.)? Please provide details of how this will be achieved.

Yes. We will be sharing a summary report to the clinics where the study was conducted, this will be accessed by the providers who participated in the study. The results will also be shared with the community advisory group and the community leadership.

**Non-human subjects research using specimens/ animals collected as part of the study, or those housed in archival collections. Examples include archaeology, paleontology, botany and zoology.**

Did the permission you obtained from a local authority to perform the study include an agreement on access to outputs and benefit sharing? This may include procedures to enable fair distribution of the benefits and resources arising from the research performed. Please include any details of Prior Informed Consent and Benefit Sharing Agreements obtained. These may be required by field-specific regulations, for example the Convention on Biological Diversity (CBD) and the associated Nagoya Protocol.

N/A

If the material used in your study was imported, please A) provide the year it was imported and B) indicate whether permits were obtained to import/export the materials used, C) provide details of any permits obtained. If this information is not available, please indicate this.

N/A

If you used archival specimens, please state how the material used in your study was acquired by the institute it is held in and provide details of any permits obtained for the original excavations/ sample collection. If this information is not available, please indicate this.

N/A

How was the potential cultural significance of the materials collected in your study to local communities considered in your research design? Were Indigenous peoples and/or local researchers and institutions involved with archaeological excavations / collection of specimens? If so, please provide a description of their involvement.

N/A

If your manuscript includes photographs of human remains please indicate whether authors obtained permission from descendants or affiliated cultural communities to do so.

N/A
